# Naxos Disease in Two Siblings

**DOI:** 10.4103/0974-7753.66917

**Published:** 2010

**Authors:** G Meera, D Prabhavathy, S Jayakumar, GK Tharini

**Affiliations:** Department of Dermatology, Madras Medical College, Chennai, India

**Keywords:** Consanguinity, woolly hair, striate palmoplantar keratoderma

## Abstract

Naxos disease is a rare cardiocutaneous disorder characterized by palmoplantar keratoderma, woolly hair and arrythmogenic right ventricular cardiomyopathy. We report two siblings with Naxos disease with right middle lobe syndrome in one of them.

## INTRODUCTION

Naxos disease is an autosomal recessively inherited familial syndrome that is characterized by woolly hair, palmoplantar keratoderma and arrhythmogenic right ventricular dysplasia. It was first reported in 1986 by Protonotarios *et al*. in patients originating from the Hellenic island of Naxos[1] The prevalence of the disease in Greek islands is 1:1000. Apart from Naxos, affected families have been detected in other Greek Aegean islands, Turkey, Israel and Saudi Arabia.[2] A variant of Naxos disease, reported as Carvajal syndrome, has been described in families from India and Ecuador. A two base-pair deletion in the plakoglobin (cell adhesion protein) gene (Pk2157del2TG), which maps to 17q21, has been identified as the cause of Naxos disease. Two different mutants of the desmoplakin gene have been found to be the cause of Carvajal syndrome.

## CASE REPORT

**Figure 1:**
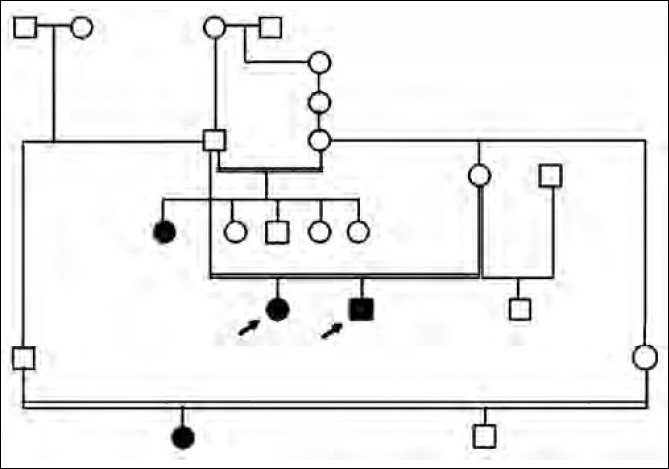
Family pedigree

Two siblings, a eleven year-old female and a eight year-old male presented with history of curly hair since birth, and thickening of palms and soles since the age of one. The female child had history of breathlessness, occasionally episodes prolonged for up to Six months. The father of the two siblings married two sisters (second degree consanguinity); one child through his first wife is affected apart from these two siblings borne through his second wife [Figure 1]. His second wife has an unaffected child through her ex-husband (nonconsanguineous marriage). A cousin of the affected siblings is also affected [Figure 1].

**Figure 2:**
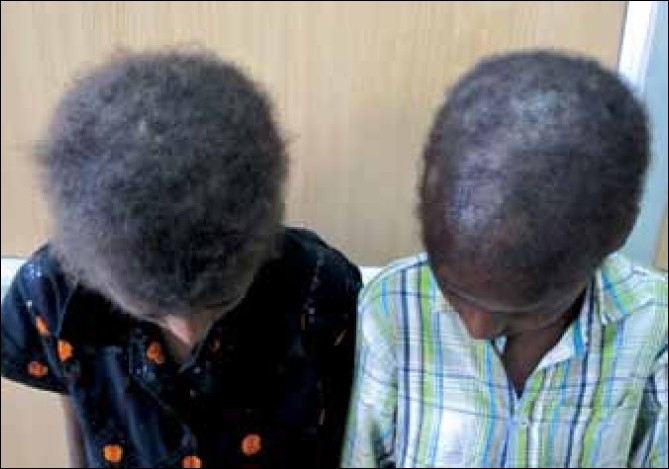
Woolly hair

**Figure 3:**
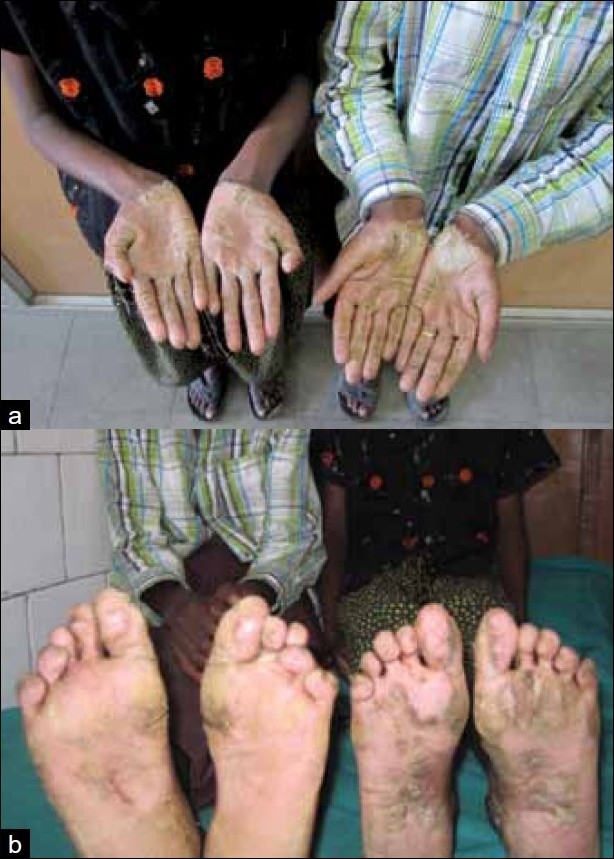
(a) Striate palmar keratoderma, (b) striate plantar keratoderma

**Figure 4:**
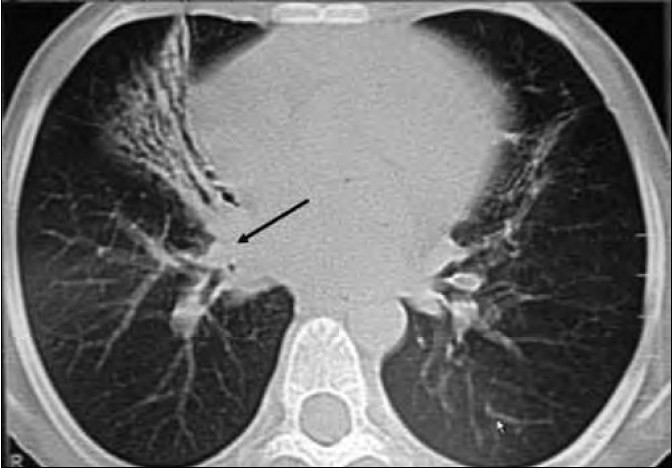
Right middle lobe syndrome

Clinical examination of the scalp, palms and soles of the siblings revealed sparse, fine, pale, curly, brittle hair having a woolly appearance [Figure 2] and longitudinal hyperkeratosis of striate form of palmoplantar keratoderma [Figure 3a and b, respectively]. The female child showed xerosis of the skin, deformed ears and clubbing of finger nails. Electrocardiogram (ECG) was done for both the children and showed inverted T waves in leads V_1_,V_2_; QTc: 533 m s and epsilon waves in the female child and inverted T waves in leads V1-V4, QTc: 490 m s and Right bundle branch block (RBBB) in male child. Echocardiography (ECHO) and hair shaft examinations were normal in both the cases. Chest X ray of the female child showed paratracheal adenopathy on the right and bilateral lower zone consolidation. Contrast Computed tomography (CT) of the chest showed bilateral midzone and lingular bronchiectasis with middle lobe syndrome on the right side [Figure 4]. Further clinical examination and investigations were within normal limits in both the children.

Based on the history, clinical features and investigations, a diagnosis of Naxos disease was made. Both siblings were given emollients; topical keratolytics and antibiotics were given whenever and wherever necessary. Patients were advised regular follow up in the cardiology and thoracic medicine departments for further management of emergent symptoms.

## DISCUSSION

Naxos disease is a rare genodermatosis characterized by congenital woolly, curly, rough and light colored scalp hair at birth, with palmoplantar keratoderma appearing over the pressure areas during the first year of life, when infants start using their extremities.[3] Case reports of Naxos disease are on the rise from India and ours is one. The cardiomyopathy clinically manifests in adolescence when patients may develop progressive heart disease involving the right or both ventricles with an adverse prognosis, especially in younger patients. Symptoms of right heart failure are found in the final stages when the right or both ventricles are severely affected. In our case report both the siblings are of prepubertal age and showed only ECG changes attributable to Naxos disease with no symptoms suggestive of cardiac involvement at present. In a long-term study on patients with Naxos disease, it was shown that risk factors for sudden death included history of syncope, the appearance of cardiac symptoms, severely progressive disease of the right ventricle before the age of 35 years and the involvement of the left ventricle.[2]

ECG abnormalities in Naxos disease included inverted T waves in leads V_1_ through V_3_ or across the precordial leads, QRS complex prolongation in leads V_1_ through V_3_, epsilon waves, and complete or incomplete right bundle-branch block. Low voltage and/or flat T waves in left precordial leads were observed mostly in severe right or biventricular involvement.[4]

In Naxos disease, cardiac histology reveals the characteristic loss of the right ventricular myocardium with fibro-fatty replacement. Defects in the linking sites of cell adhesion proteins can interrupt the contiguous chain of cell adhesion, particularly under conditions of increased mechanical stress or stretch, leading to cell death, progressive loss of myocardium and fibro-fatty replacement.

The primary goal is the prevention of sudden death. Implantation of an automatic cardioverter defibrillator is indicated in patients who develop symptoms and/or structural progression, particularly before the age of 35 years.[5] Antiarrhythmic drugs are indicated for preventing recurrences of episodes of sustained ventricular tachycardia. In an attempt to control Naxos disease, systematic genetic screening of the populations at risk has been initiated and is starting to identify the heterozygous carriers of the plakoglobin gene mutation.

Right middle lobe syndrome (RMLS)[6] generally refers to atelectasis in the right middle lobe of the lung. Certain anatomical characteristics make the right middle lobe susceptible to transient obstruction as a result of inflammation or edema. The narrow diameter of the lobar bronchus and acute take-off angle create poor conditions for drainage. Relative anatomical isolation of the middle lobe and poor collateral ventilation decrease the chance of reinflation once atelectasis occurs. Bronchial obstruction can result from extrinsic compression as in hilar lymphadenopathy or tumor of neoplastic origin. Management is determined by etiology, and most patients respond to medical therapy alone. Right middle lobe syndrome is essentially a radiographic diagnosis, and physical findings widely vary. Prompt diagnosis and initiation of medical therapy including the administration of antibiotics and the avoidance of irritating agents may be effective. However, abnormal shadows on chest radiography remain unchanged even when acute symptoms have disappeared, suggesting latent lesional inflammation or recurrence.

Thus, Naxos disease, a rare occurrence by itself, the occurrence of right middle lobe syndrome in one of the siblings is still a rarer finding. Further case reports are needed to assess the incidence of right middle lobe syndrome in patients with Naxos disease.

Whenever woolly hair is associated with any kind of palmoplantar keratoderma, a search for possible cardiac abnormalities is recommended. A regular follow-up and treatment at the time of symptoms will prevent sudden death in these patients.
